# Infiltrative gliomas of the thalamus in children: the role of surgery in the era of H3 K27M mutant midline gliomas

**DOI:** 10.1007/s00701-020-04589-y

**Published:** 2020-10-22

**Authors:** Christian Dorfer, Thomas Czech, Johannes Gojo, Arthur Hosmann, Andreas Peyrl, Amedeo A. Azizi, Gregor Kasprian, Karin Dieckmann, Mariella G. Filbin, Christine Haberler, Karl Roessler, Irene Slavc

**Affiliations:** 1 Department of Neurosurgery, Medical University of Vienna, Währinger Gürtel 18-20, 1090 Vienna, Austria; 2 Comprehensive Cancer Center-CCC CNS Unit, Medical University of Vienna, Vienna, Austria; 3 Department of Pediatrics and Adolescent Medicine, Medical University of Vienna, Vienna, Austria; 4 Department of Biomedical Imaging and Image-Guided Therapy, Medical University of Vienna, Vienna, Austria; 5 Department of Radiotherapy, Medical University of Vienna, Vienna, Austria; 6 Department of Pediatric Oncology, Dana-Farber Boston Children’s Cancer and Blood Disorders Center, Boston, MA USA; 7 Division of Neuropathology and Neurochemistry, Department of Neurology, Medical University of Vienna, Vienna, Austria

**Keywords:** Thalamic tumor, Diffuse midline glioma, Pediatric brain tumor

## Abstract

**Background:**

The role of surgery in the management of pediatric non-pilocytic infiltrative thalamic gliomas needs to be revisited specifically with regard to molecularly defined subtypes.

**Methods:**

A retrospective review of a consecutive series of children operated on a thalamic tumor between 1992 and May 2018 was performed. Neuroimaging data were reviewed for localization and extent of resection; pathology was re-reviewed according to the current WHO classification, including assessment of histone H3 K27 mutational status.

**Results:**

Forty-nine patients with a thalamic tumor aged < 18 years at diagnosis were identified. Twenty-five patients (51%) had a non-pilocytic infiltrative glioma, of which the H3 K27M status was available in 22. Fourteen patients were diagnosed as *diffuse midline glioma (DMG) H3 K27M mutant*. There was no statistically significant difference in survival between patients harboring the H3 K27M mutation and wildtype. Resection (“any resection > 50%” vs “biopsy”) and histological tumor grade (“°II” vs “°III+°IV”) were statistically significant predictors of survival (univariate: p = 0.044 and p = 0.013, respectively). These results remained significant on multivariate analysis (HR 0.371/*p* = 0.048, HR 9.433/*p* = 0.035).

**Conclusion:**

We advocate to still consider an attempt at maximal safe resection in the multidisciplinary treatment of unilateral thalamic non-pilocytic gliomas irrespective of their H3 K27-mutational status.

## Introduction

Thalamic tumors are rare neoplastic lesions representing about 2–5% of all pediatric brain tumors [12, 31, 37]. Most neoplasms in this location are of glial lineage, with up to 50% showing low-grade histology [16]. The predominant low-grade histology in children represents pilocytic astrocytoma with its characteristically well-circumscribed, contrast-enhancing, solid-cystic appearance on MRI that distinguishes it from other non-pilocytic histologies with often less-defined margins, no or inhomogeneous contrast enhancement and necrosis [8, 17, 28, 30].

The 2016 revision of the World Health Organization (WHO) classification of tumors of the central nervous system (CNS) defined *diffuse midline glioma (DMG), H3 K27M mutant* as a new tumor entity corresponding to WHO grade IV independent of histological signs of anaplasia. The updated WHO classification thereby combines all infiltrating gliomas harboring the same canonical mutation at the Lysine 27 of the histones H3 tail and arising in the pons, thalamus, and spinal cord into one entity [24, 26]. This mutation was identified as the most important predictor of worse outcome in thalamic tumors regardless of histology [32].

Over the last two decades, technical advances including multimodal imaging, neuronavigation, intraoperative imaging, and electrophysiological monitoring have led to a shift from stereotactic biopsy of thalamic tumors towards an attempt at maximal safe resection [2–5, 7, 10, 12, 17, 20, 27, 29, 30, 36]. These technologies allow us to accurately depict the anatomical boundaries and extension of the thalamic lesions, to visualize critical white matter tracts by diffusion tensor imaging (DTI), and to use this information with the neuronavigational systems when operating under direct neurophysiologic monitoring [11, 21, 27].

The release of the 2016 revision of the WHO classification with the definition of one tumor entity to describe diffuse midline gliomas, H3 K27M mutant, irrespective of their specific location along the midline as well as histologic features now challenges the perception that maximal safe resection is still the aim in thalamic tumors. It specifically ignores the fact that counseling parents at the time of presentation could significantly differ between the thalamic and pontine location.

Here, we present our experience in the management of non-pilocytic pediatric thalamic gliomas, and discuss the role of surgery in the “H3 K27M era.”

## Patients and methods

### Clinical data

All patients that were operated at the Department of Neurosurgery, Vienna Medical University, since 1992 have been prospectively entered into a database and were available for retrospective analysis. We created a datasheet from a medical chart review for a consecutive series of children operated on a thalamic tumor. Magnetic resonance imaging (MRI) data were reviewed for anatomic location and extent of resection (EOR). Tumors primarily localized in the basal ganglia, cerebral white matter, hypothalamus, pineal gland, or optic pathway were excluded. The degree of resection was defined as gross total resection (GTR) (100%), as subtotal (STR) (> 90%) and partial resection (PR) (50–90%).

We used neuronavigational system since 1997 (Easy Guide Ga. Phillips 1997–2003 and Medtronic Navigation from 2003 onwards). Currently, we apply T1, T2, and DTI images as well as a venous CTA, if performed. Intraoperative neurophysiological monitoring (including somatosensory evoked potentials, transcranial motor evoked potentials, and subcortical stimulation mapping) was used in all 22 cases that underwent surgical resection since 2002.

Adjuvant postoperative treatment with concomitant chemo-and local radiotherapy followed by consolidation therapy was administered according to the HIT-GBM-C [41], HIT-GBM-D [42], or HIT-HGG-2007 (EudraCT 2007-000128-42) trial protocols. One patient initially diagnosed as PNET was treated within the HIT 2000 trial with craniospinal radiotherapy and maintenance chemotherapy (14). One patient was treated with chemotherapy only according to the HIT-LGG-1996 protocol (15).

### Pathology

All tumors were re-reviewed by an experienced neuropathologist (C.H.) and classified according to the 2016 revision of the WHO classification of tumours of the central nervous system [24–26]. Tumors classified as pilocytic astrocytoma (PA) and other WHO grade I tumors were excluded from further detailed analysis. Tumors with available FFPE tissue were stained with rabbit polyclonal antibodies against H3 K27me3 (07-449 Millipore) and H3 K27M mutant (ABE419 Millipore). Tumors displaying nuclear expression of H3 K27M mutant and loss of H3 K27me3 were considered harboring a *H3 K27M* mutation.

### Statistical analysis

Data are presented as total counts and percentage or as median values and data range for continuous parameters. Overall survival (OS) was estimated using Kaplan-Meier analysis. Predictors of overall survival were identified using log rank test (for univariate analysis). For multivariate analysis, significant values of the univariate analysis were included in the Cox proportional hazard model. Differences were considered to be statistically significant at a two-sided *p* value of < 0.05. Statistical analysis was performed using SPSS® Statistics 22 (IBM Corp., Armonk, NY).

The study was approved by the Ethics Committee of the Medical University of Vienna. (EC-Nr.: 933/2010)

## Results

Between March 1992 and May 2018, 49 patients with a thalamic tumor aged < 18 years at diagnosis were identified. The median age at diagnosis was 10.0 years (range 2–18 years). Twenty-two patients (45%) had a pilocytic astrocytoma (WHO °I) and a WHO grade II–IV tumor was diagnosed in 27 children (55%).

All 22 patients with a pilocytic astrocytoma are alive after a median follow-up time of 10.9 years. Two of 27 patients with a non-pilocytic tumor had an atypical teratoid-rhabdoid tumor (ATRT) and are alive after intensified chemotherapy and focal radiotherapy for 7 and 14 years [33].

A glioma grade II–IV was diagnosed in 25 patients (Table 1). In 3/25 patients, H3 K27M evaluation was not possible because no tumor tissue was available for further analysis. A bithalamic tumor was found in 7/25 patients.

**Table 1:** Infiltrative gliomas—patient’s characteristics

Patient No.	Age at diagnosis/sex	H3K27 status	Diagnosis (WHO 2016)	Histological features^a^	Localization	Degree of resection	Adjuvant therapy	Follow-up time (years)	Follow-up status
1	3/F	Mutated	DMG *H3K27mut*	Astrocytoma II	Thalamic l	STR	CTX/RTX^b,c^	9.6	DOD
2	15/F	Mutated	DMG *H3K27mut*	Oligodendroglioma III	Thalamic r	STR	CTX/RTX^c^	9.2	CR
3	4/F	Mutated	DMG *H3K27mut*	Astrocytoma II	Thalamic r	STR	CTX/RTX^c^	4.0	PD
4	10/F	Mutated	DMG *H3K27mut*	Astrocytoma III	Thalamic l	Biopsy	CTX/RTX^e^	1.5	DOD
5	9/M	Mutated	DMG *H3K27mut*	Astrocytoma III	Thalamic r	STR	CTX/RTX^e^	1.1	DOD
6	5/F	Mutated	DMG *H3K27mut*	Astrocytoma III	Bithalamic	Biopsy	CTX/RTX^c^	1.1	DOD
7	13/F	Mutated	DMG *H3K27mut*	Astrocytoma III	Thalamic r	Biopsy	CTX/RTX^c^	0.8	DOD
8	5/M	Mutated	DMG *H3K27Mmut*	Astrocytoma III	Bithalamic	Biopsy	CTX/RTX^d^	0.6	DOD
9	8/M	Mutated	DMG *H3K27Mmut*	GBM	Thalamic l	STR	CTX/RTX^c^	2.0	DOD
10	9/M	Mutated	DMG *H3K27mut*	GBM	Thalamic l	STR	CTX/RTX^c^	1.6	DOD
11	8/M	Mutated	DMG *H3K27mut*	GBM	Thalamic l	STR	CTX/RTX^c^	1.2	DOD
12	9/F	Mutated	DMG *H3K27mut*	GBM	Thalamic l	PR	CTX/RTX^e^	0.9	DOD
13	9/F	Mutated	DMG *H3K27Mmut*	GBM	Thalamic r	PR	CTX/RTX^c^	0.5	DOD
14	17/F	Mutated	DMG *H3K27mut*	GBM	Thalamic r	PR	RTX	0.4	DOD
15	11/M	Wild-type H3K27me3-loss	GBM	GBM	Thalamic l	PR	CTX/RTX^c^	1.5	PD
16	5/M	Wildtype; H3K27me3-loss	AA,NOS	Astrocytoma III	Bithalamic	Biopsy	CTX/RTX^e^	0.9	DOD
17	15/F	Wildtype	AA , IDHwt	Astrocytoma III	Bithalamic	PR	CTX/RTX^c^	7.6	SD
18	11/M	Wildtype	AA, IDHwt	Astrocytoma III	Bithalamic	Biopsy	CTX/RTX^c^	1.2	DOD
19	17/M	Wildtype	AA, IDHwt	Astrocytoma III	Thalamic r	STR	CTX/RTX^f^	0.9	DOD
20	4/M	Wildtype	AA, IDHwt	Astrocytoma III	Thalamic l	Biopsy	CTX/RTX^c^	0.7	DOD
21	7/F	Wildtype	LGG, NOS	Astrocytoma II	Thalamic r	PR	-	n/a	LTFU
22	9/M	Wildtype	LGG, NOS	Astrocytoma II	Bithalamic	PR	CTX^g^	20.0	SD
23	5/M	n/a	Diffuse A, NOS	Astrocytoma II	Thalamic l	Biopsy	CTX/RTX^d^	4.0	DOD
24	15/M	n/a	AA, , NOS	Astrocytoma III	Thalamic l	PR	CTX/RTX^d^	3.0	DOD
25	18/M	n/a	Anaplastic oligoastrocytoma, NOS	Oligoastrocytoma III,	Bithalamic	Biopsy	CTX/RTX^c^	2.0	DOD

*AA*, anaplastic astrocytoma; *CTX*, chemotherapy; *DMG*, diffuse midline glioma; *DOD*, death of disease; *GBM*, glioblastoma; *LGG*, low-grade glioma; *LTFU*, lost to follow-up; *NOS*, not otherwise specified; *STR*, subtotal resection; *PR*, partial resection; *RTX*, radiotherapy; *SD*, stable disease; *M*/*F*, male/female; *r*/*l* right/left

^a^As defined by the 2007 4th edition of the WHO classification of tumours of the central nervous system (25). ^b^At recurrence 9 years after primary diagnosis. ^c^HIT-HGG-2007. ^d^HIT-GBM-C (41). ^e^HIT-GBM-D (42). ^f^HIT 2000 (14). ^g^HIT-LGG-1996 (15)

Twenty-eight surgical procedures were performed in these 25 patients. Stereotactic biopsy only was used in 9 patients. Twenty resective surgeries were performed in the remaining 16 patients with two surgeries for recurrence and one staged surgery. Open resection was planned only if a safe major debulking > 50% seemed achievable. Surgical approaches included anterior transcallosal (10), supracerebellar-infratentorial (3), combined parieto-occipital transcortical + interhemispheric transtentorial (2), occipital interhemispheric para/subsplenial (1), transsylvian (1), parieto-occipital interhemispheric transtentorial (1), parietal transcortical (1), and posterior transcallosal (1). Subtotal resection (> 90%) was achieved in 8 patients.

There was no operative mortality. Transient postoperative neurological morbidity included diplopia (2), visual field defect (1), slowing of speech (1), worsening of a hemiparesis (3), and of an ataxia in two patients. A permanent new visual field defect was present in 2 patients, and worsening of hemiataxia and of hemiparesis in one patient each.

Twelve of the 25 patients (48.0%) had a ventriculo-peritoneal shunt implanted at some time point of their treatment.

The median follow-up time (as of January 2020) was 1.4 years (range 0.4–20 years). One patient was lost to follow-up.

**Fig. 1:**
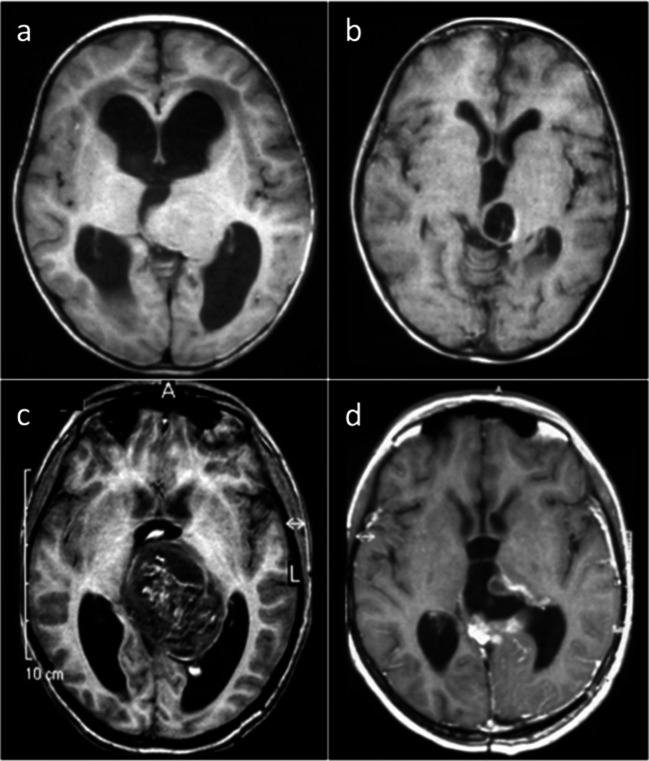
Case1 (a) axial T1-weighted MRI depicting a left thalamic tumor, (b) stable tumor remnant 5 years after STR via a suboccipital-transtentorial approach, (c) 9 years after diagnosis rapid progression as GBM with intratumoral bleeding, (d) early postoperative MRI after a combined occipital-transcortical and suboccipital-transtentorial STR, the patient rapidly progressed despite chemotherapy/radiotherapy

**Fig. 2:**
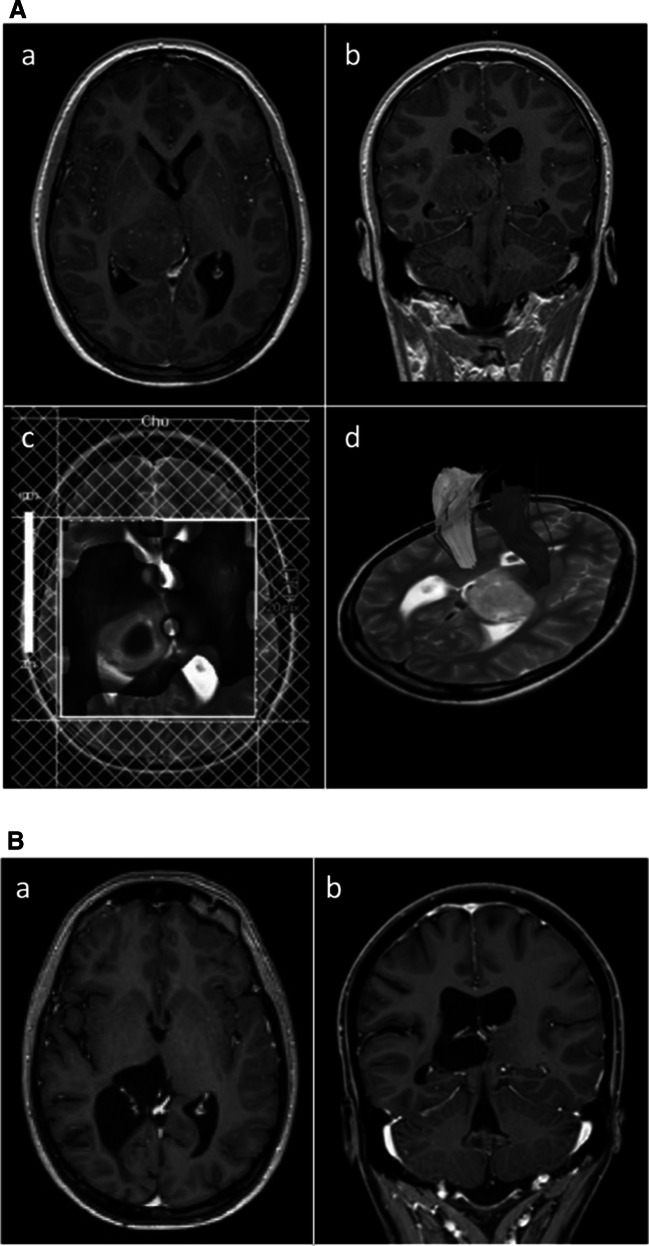
(A) Case 2 (a) axial and (b) coronal T1-weighted contrast-enhanced MRI depicting a right posterior thalamic tumor, (c) multivoxel spectroscopy indicating high cellular turnover, (d) DTI depicting the posterior limb of the internal capsule displaced anterolaterally. (B) Case 2 (a) axial and (b) coronal T1-weighted contrast-enhanced MRI at 9 year follow-up after STR via a posterior interhemispheric-subsplenial/parasplenial approach, chemo- and radiotherapy

Fourteen patients were diagnosed as “diffuse midline glioma (DMG), H3 K27M mutant”. Out of these, 5 patients (cases 4–8) with initial histology anaplastic astrocytoma WHO °III died with progressive disease at a median of 1.1 years (range 0.6 to 1.5) after surgery followed by radio- and chemotherapy. Glioblastoma (GBM) WHO °IV was initially diagnosed in 6/14 patients (cases 9–14). After undergoing extensive resection, and radio- and chemotherapy, the patients died at a median of 1.0 year (range 0.4 to 2.0). One of these patients, who had an additional BRAF(V600E) mutation, additionally received bevacizumab and a combined BRAF-/MEK inhibitor at progression and survived for 25 months (case 9). Three of the 14 patients with a DMG H3 K27M mutant had a survival over 2 years (cases 1–3). The extent of resection was STR in all three. Two of these had cytomorphological features of an astrocytoma WHO °II (cases 1 and 3) and one of an anaplastic oligodendroglioma WHO °III (case 2). Case 1 with a diffuse astrocytoma WHO °II had stable disease without further treatment for 9 years before the tumor progressed rapidly as GBM, and the patient died within a few months despite another STR followed by chemotherapy and radiotherapy (Fig. 1). Case 2 underwent STR, radiotherapy, and chemotherapy and is alive and in complete remission at 9.2 years (Fig. 2). Case 3 had radiotherapy and temozolomide after a staged PR/STR resection, and underwent one re-resection 3.4 years later with a follow-up of 4 years.

Of the remaining 8/22 diffuse gliomas with H3 K27M status available, 2/8 had no *H3 K27M* mutation but loss of tri-methylation, which seems to translate into a similar worse prognosis [9]. One of these had a bithalamic tumor (astrocytoma WHO °III) and died within 1 year after biopsy, chemotherapy, and focal radiotherapy. The second patient had a biopsy (GBM) followed by chemotherapy and radiotherapy at another institution, underwent an extensive PR 4 months later, and is now progressive under targeted therapy plus temozolomide at 18 months.

Four H3 K27M wild-type patients (cases 17–20) had an anaplastic astrocytoma WHO °III. Three of them died at a median of 0.9 years (range 0.7 to 1.2) after undergoing resection in one and biopsy in two cases followed by chemotherapy and radiotherapy. The remaining patient had a bithalamic tumor and has stable disease for 7.6 years after partial resection followed by chemotherapy and focal radiotherapy.

Two H3 K27M wild-type patients (cases 21, 22) had a diffuse astrocytoma, NOS; both underwent partial resection. One patient with a bithalamic tumor received additional chemotherapy consisting of carboplatin and vincristine according to the HIT LGG96 protocol [14] and is alive at 20 years, and one was lost to follow-up.

From the three patients with no H3 K27M status available (patients 23-25), one had a diffuse low-grade astrocytoma and survived for 4 years, one had an anaplastic astrocytoma and survived for 3 years, and one who had a bithalamic anaplastic oligoastrocytoma survived for 2 years. All three were treated with chemotherapy and focal radiation.

**Fig. 3:**
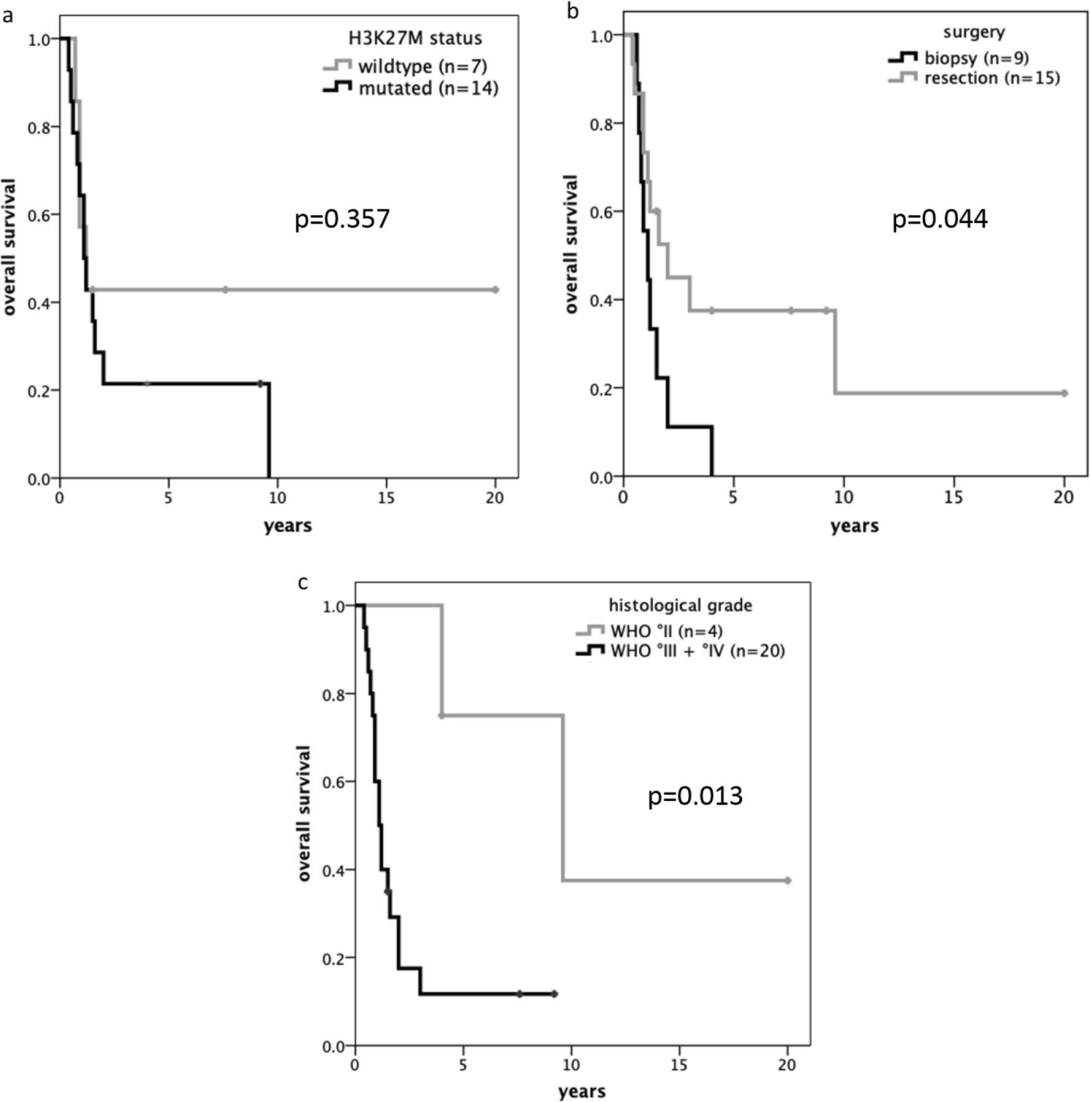
Mutational status does not predict survival (a); EOR (b) and histological grade (c) are positive predictors for survival

For the whole cohort, there was no statistically significant difference in survival between patients harboring the H3 K27M mutation and wildtype. EOR (“any resection > 50%” vs “biopsy”) and histological tumor grade (“°II” vs “°III+°IV”) were statistically significant predictors of survival (Fig. 3). These results remained significant on a multivariate analysis that included H3K27 mutational status, EOR, and histological grade: “biopsy” vs “any resection > 50%” (HR 0.371/*p* = 0.048) and “°II” vs “°III+°IV” (HR 9.433/*p* = 0.035).

## Discussion

We reviewed our institutional experience of the surgical management and outcome of 25 pediatric patients with an infiltrative glioma WHO °II–°IV of the thalamus in the MRI era taking into account the current molecular classification of these tumors (24). This cohort constitutes 50% of a consecutive series of thalamic tumors in the pediatric age group operated on at our institution. A major debulking procedure (EOR > 50%) was achieved in 16 patients. A stereotactic biopsy only was performed in 9 patients. Resective surgery was a positive predictive factor for survival. Fourteen patients (56%) were diagnosed as a “diffuse midline glioma, H3 K27M mutant”, a tumor category defined by the 2016 revision of the WHO classification, and graded °IV irrespective of traditional histopathological criteria (24,26). We did not find a statistically significant difference in survival between patients harboring the H3 K27M mutation and wildtype. Of note three patients with a H3 K27M mutated tumor had a long survival.

In 1984 Bernstein et al. reported on the 30-year Toronto experience with thalamic tumors and recommended “partial resection if deemed safe” [4]. The same group later corroborated this opinion specifically for low-grade gliomas where they achieved an EOR > 50% in a more recent (1976–1991) series of 13 children, with two-thirds being fibrillary non-pilocytic astrocytomas [18]. With technical advances improving the planning and safety of the surgical intervention, the general attitude has shifted to consider maximal safe resection in selected cases instead of a biopsy only approach.

With pilocytic astrocytomas comprising approximately one-third of these tumors [15], the safety of this strategy in tumors with a histologically and surgically identifiable brain-tumor interface has been reported by several experienced centers [3, 5, 7, 8, 10, 13, 27, 29, 30].

The role of resective surgery is less clear for non-pilocytic infiltrative gliomas. Even though the correlation between extent of resection and prognosis has been reported for this localization too [22, 32], acknowledging the potential risks for neurological worsening as well as the dismal prognosis for the majority of these tumors, there is currently no consensus on the surgical approach [2, 10, 19, 22, 23, 37].

Also the majority of the reported experience does not specifically address the role of resective surgery in the subgroup of diffuse gliomas by including not only PA and other WHO°I tumors but also other non-glioma entities into their analysis. Recent single-institution series of surgically treated pediatric patients with thalamic tumors are summarized in Table 2. Only those pediatric patients with histologically confirmed unilateral glial tumors were taken into account. Although there is a high percentage of WHO°I tumors (mostly pilocytic astrocytoma) in all series, an EOR > 90% could be achieved in a high number of tumors with acceptable although non-negligible surgical risks.

**Table 2:** Literature review of single-center series of surgically treated unilateral thalamic gliomas since 1997

Authors and year	Treatment period	N gliomas^a^/total	Histology	EOR	Surgical mortality	Permanent neurological worsening
Cuccia/Monges, 1997	1988–1994	24/26	9 LGG, 15 HGG	GTR 9/26, PR (40–90%) 10/26, B (< 30%) 7/26	2	4 (motor3/consc.1; *trans./perm.?*)
Reardon, 1998	1985–1996	24/36	9 PA, 2 GG, 13 °II	> 90% 7/36, 50–90% 7/36	*n.d.*	*n.d.*
Steiger, 2000	?	5/14	2 PA,3 ° III	GTR 2 (PA), > 80% 3 (°III)	0	1 (visual)
Özek, 2002	1992–	14/18	11 LGG, 3 HGG	GTR 16/18	0	*n.d.*
Albright, 2004	1986–2001	19/19	5 PA, 2 °II, 7 °III, 5 °IV	GTR 6 > 90% 16	1	2 (motor, consc.)
Fernandez, 2006	1984–2004	14/14	5 PA, 1 °II, 6 °III, 2 °IV	GTR 2 B 3	0	1 (coma)
Baroncini 2007	1992–2003	11/16	4 PA, 2 °II, 4 °III, 1 °IV	GTR 5 NTR (< 1.5cm^3^) 2	0	4 (visual)
Puget, 2007	1989–2003	48/69	14 °I (13 PA,1 GG), 13 °II, 21 HGG	GTR 5/54^b^ STR (> 90%) 22/54^b^ B 19/54^b^	2	16 (*trans./perm.?*)
Bilginer, 2014	1999–2012	29/45	15 °I (14 PA, 1GG), 3 °III, 11 °IV	GTR 6/33^c^ STR 21/33^c^ PR (40-90%) 6/33^c^	0	0
Cinalli, 2018	2002–2016	23/27	12 PA, 4 GG, 1 °II, 1 °III, 5 °IV	GTR 12/23 STR (> 90%) 5/23	1 (2)	2 (motor+aphasia)

*B*, biopsy; *consc.*, consciousness; *EOR*, extent of resection; *GG*, ganglioglioma; *GTR*, gross total resection; *HGG*, high-grade glioma; *LGG*, low-grade glioma; *n.d.*, no details; *NTR*, near-total resection; *PA*, pilocytic astrocytoma; *perm.*, permanent; *PR*, partial resection; *STR*, subtotal resection; *trans.*, transient

^a^Excluding patients either > 18 years of age, or with bithalamic tumors, or with non-glial histology, or without histological diagnosis

^b^54 unilateral thalamic tumors operated on (all histologies)

^c^33 unilateral thalamic tumors operated on (all histologies)

Boesten et al. reported on pediatric patients with thalamic low-grade tumors treated within the multicentric HIT-LGG 1996 and SIOP-LGG 2004 trials between 1996 and 2012 [6]. Within the subgroup of 73 histologically confirmed monothalamic glial tumors, 20 (27%) were neither pilocytic astrocytomas (65%) nor other WHO °I glial tumors (7%). GTR or STR (< 1.5 cm^3^) was reported in only 12 patients (14%) of the whole group; no information on surgical morbidity is provided.

In case of a diffuse glioma in the thalamic region, one ultimately has to question the impact of intentionally going for a less than “radical” (< 90%) resection, as only this EOR has been shown to confer a statistically significant survival benefit in malignant gliomas of childhood [40]. In our series for the 16 patients considered for a resective procedure, an EOR > 90% and an EOR 90–50% were achieved in eight patients each. The localization-dependent potential surgical morbidity when striving for a more than reasonable resection in these tumors obviously will impact on the oncological outcome. This could be a potential bias in multicentric trial reports. When compared with biopsy, only our resection group did better, also on a multivariate analysis including the H3 K27M status. Neither our data nor those reported in the literature allow for a strong opinion on the overall impact of more limited resections.

Two retrospective multicentric studies based on patients enrolled in various HIT-GBM trials between 1997 and 2007, [22] and recently with additional data from the HIT-HGG-2007 trial included only patients with centrally reviewed histologically defined high-grade gliomas (HGG) [19].

In Kramm’s study with subtotal or partial resection performed in ~ 50% of the 99 patients analyzed, EOR (“resection > 50%” vs “no resection”) and histological grading (°III vs °IV) were identified as independent prognostic factors for OS and EFS. No information is available on surgical complications nor on the number of bithalamic tumors [22].

Karremann et al. analyzed the impact of H3 K27 phenotype, grading, localization of the tumor, and EOR in cohort of 77 patients aged > 3 years at diagnosis with a HG diffuse glioma of the craniospinal midline [19]. Within the group of 32 tumors of the “thalamic region” (including thalamic and basal ganglia), 20 patients underwent a > 50% resection, only 4 of them > 90%. With 24 H3 K27M mutated vs 8 wild-type thalamic HG tumors, the H3 K27 phenotype was the only risk factor for OS, although the authors do not state if this was valid for univariate analysis only. While survival was not affected by EOR in the entire cohort (including 11 spinal and 7 “other midline” HGG in addition to 27 DIPGs), there was a trend towards improved OS in the four H3 K27 wild-type gliomas with > 90% resection, with only one thalamic tumor. No information is provided on any potential impact of EOR > 50% vs less. No data on surgical complications were provided. Nevertheless, based on their results, the authors definitely cautioned against an attempt at resection in the presence of a H3K27M mutation.

Recently Gutierrez et al. analyzed 113 pediatric non-brainstem high-grade gliomas treated within the multicentric Herby trial [17]. Within the group of 45 “midline” gliomas, 31 patients had a strictly unilateral thalamic localization^1^. Regarding EOR, survival in the whole cohort including 68 hemispheric tumors was statistically significantly correlated with a resection > 95%. For the unilateral thalamic tumors, resection > 50% was reported in 52% (16/31); no information on surgical morbidity is provided. The H3 K27 mutational status assessed in 32 of the 45 “midline” tumors did not correlate with survival.

An argument in favor of maximal safe tumor removal instead of biopsy only is that histological features and grading throughout the tumor should not be completely dismissed even in H3 K27M mutant thalamic tumors [6, 34]. Generalizing and dichotomizing these tumors strictly into H3 K27M mutant and non-mutant without considering histological features and WHO grading may pose the risk of misconceptions regarding tumor biology and expected prognosis. In our series, two patients with H3 K27M mutant tumors, graded WHO II, survived for 9.6 and 4 years. Two patients °II were wild-type tumors, with one of them still alive at 20 years, the other being lost to follow-up. These numbers do not allow any robust inferences on how the histopathological feature “grade II” impacts on survival in H3 K27M mutant tumors. Lumping the °II and °III category together in our series, an OS benefit was present in this group vs GBM. This could indicate that in non-GBM H3 K27M mutant tumors every attempt at a good functional long-term outcome is justified. Also in H3 K27M mutant thalamic GBM patients targeted personalized therapies may improve outcome. In our series, one H3 K27M mutant GBM patient who had an additional BRAF(V600E) mutation survived for 2 years with bevacizumab and combined BRAF-/MEK inhibitor targeted therapy.

Steinbok et al. reported on the multicentric Canadian experience treated in the MRI era 1989–2012 [37]. Histology was available in 42/62 patients with unithalamic tumors, with 33 glial tumors. Out of these only 17 had a diagnosis of diffuse glioma, with 13 high-grade lesions. With EOR dichotomized as “> 95%” vs “< 95%,” the difference in 5-year OS was found to be statistically significant (79.8% vs 52%), although this did not hold on multivariate analysis, with only tumor grade (“low” vs “high”) remaining a predictive factor for survival. With no surgical mortality and permanent neurological worsening reported in 7/42 patients undergoing surgery, the authors are still cautious about advising to strive for GTR, with maximal safe resection as the reasonable goal [38].

Adding to this experience, Ryall et al. analyzed the Toronto experience from 1986 to 2014 with regard to the impact of H3 K27M mutation and MAPK pathway aberrations [32]. In 64 patients with a unilateral thalamic glioma, EOR (“any resection” vs “biopsy”), histological grade (“LG” vs “HG”), and H3 K27M status were significant independent predictors of survival. These results were confirmed in a validation cohort of 16 patients from Steinbok’s series, although that included 6 PA. Tumor grade and H3 K27 status were conserved as prognostic markers with removal of the PA and gangliogliomas from the Toronto low-grade cohort in univariate analysis (16 LG/22 HG). By dichotomizing EOR into “any resection” vs “biopsy,” the data provided do not allow comparison to other reports. Based on data indicating a similar biology and outcome of diffuse midline gliomas irrespective of their location in the presence of a histone H3 K27M mutation, thalamic tumors are currently often discussed together with diffuse pontine gliomas, for which surgical resection is not an option [9, 19, 39]. However, although the trend towards a worse prognosis of H3K27M mutant pediatric thalamic tumors is reported in the literature, the degree to which this quantitatively prognosticates disease severity remains unclear and the surgical management should not be generalized [1, 2, 19, 32, 35]. We show that maximal safe tumor resection should be considered also in H3 K27M mutant thalamic tumors and that long-term survival is possible.

Limitations of the study are its retrospective nature and numbers too small to draw valid statistical conclusions. This seems particularly relevant regarding survival outliers in both the H3 K27M mutant and the wild-type group. As it is unlikely that any large series will be reported comparing survival of children randomized to receive either biopsy or extensive resection, hopefully a future meta-analysis will provide more convincing data.

## Conclusion

Although current data suggest a H3 K27M mutation as a molecular signature associated with a poor prognosis in so-called diffuse midline gliomas to outweigh the impact of tumor location, we think this to be premature specifically when considering the role of resective surgery. Based on our institutional review and adding our experience to reported surgical series in experienced centers, we would still advocate to consider an attempt at maximal safe resection in the multidisciplinary treatment also of thalamic infiltrative non-pilocytic gliomas.

## Abbreviations

AA: Anaplastic astrocytoma
ATRT: Atypical teratoid-rhabdoid tumor
B: Biopsy
CNS: Central nervous system
CR: Complete remission
CTA: Computerized tomographic angiography
CTX: Chemotherapy
DOD: Dead of disease
DTI: Diffusion tensor imaging
DIPG: Diffuse intrinsic pontine glioma
DMG: Diffuse midline glioma
EFS: Event-free survival
EOR: Extent of resection
FFPE: Fresh-frozen paraffin embedded
GBM: Glioblastoma multiforme
GG: Ganglioglioma
GTR: Gross total resection
HG: High-grade
HGG: High-grade glioma
HIT: German pediatric brain tumor network
HR: Hazard ratio
LG: Low-grade
LGG: Low-grade glioma
LTFU: Lost to follow-up
MRI: Magnetic resonance imaging
NOS: Not otherwise specified
OS: Overall survival
PA: Pilocytic glioma
PR: Partial resection
RTX: Radiotherapy
SD: Stable disease
SIOP: Société Internationale Oncologie Pédiatrique
WHO: World Health Organization

## Footnotes

1 Extracted from the raw data provided by the authors, supplementary table1 [17]
